# Quantitative measurement of HER2 expression in breast cancers: comparison with ‘real-world’ routine HER2 testing in a multicenter Collaborative Biomarker Study and correlation with overall survival

**DOI:** 10.1186/s13058-015-0543-x

**Published:** 2015-03-18

**Authors:** Denise A Yardley, Peter A Kaufman, Weidong Huang, Lea Krekow, Michael Savin, William E Lawler, Stephen Zrada, Alexander Starr, Harvey Einhorn, Lee S Schwartzberg, John W Adams, Yolanda Lie, Agnes C Paquet, Jeff Sperinde, Mojgan Haddad, Steve Anderson, Marlon Brigino, Rick Pesano, Michael P Bates, Jodi Weidler, Linda Bosserman

**Affiliations:** Sarah Cannon Research Institute, 3322 West End Avenue, Nashville, TN 37203 USA; Tennessee Oncology, PLLC, 250 25th Avenue North, Nashville, TN 37203 USA; Dartmouth Hitchcock Medical Center, 1 Medical Center Drive, Lebanon, NH 03766 USA; Monogram Biosciences, Inc., 345 Oyster Point Boulevard, South San Francisco, CA 37203 USA; Texas Oncology Bedford, 1615 Hospital Parkway, Bedford, TX 76022 USA; Texas Oncology and Medical City, 7777 Forest Lane, Dallas, TX 75230 USA; St. Jude Heritage Medical Group, 2720 Harbor Boulevard, Fullerton, CA 92835 USA; The Center for Cancer and Hematologic Disease, 1930 New Jersey 70 (East), Cherry Hill, NJ 08003 USA; Monroe Medical Associates, 71 Ald Taylor Way, Harvey, IL 60426 USA; Swedish American Regional Medical Center, 1401 East State Street, Rockford, IL 61104 USA; The West Clinic, 100 N Humphreys Boulevard, Memphis, TN 38120 USA; Arlington Cancer Center, 906 West Randol Mill Road, Arlington, TX 76012 USA; Center for Molecular Biology and Pathology, Laboratory Corporation of America, Inc, Research Triangle Park, NC 27709 USA; Wilshire Oncology Medical Group, 8283 Grove Avenue, Rancho Cucamonga, CA 91730 USA; Present address: Institut de Pharmacologie Moléculaire et Cellulaire-IPMC, Sophia Antipolis, 660 Route des Lucioles, 06560 Valbonne, France; Present address: HealthTell, 3130 Crow Canyon Place, San Ramon, CA 94583 USA; Present address: Quest Diagnostics, 33608 Ortega Highway, San Juan Capistrano, CA 92675 USA; Present address: Cepheid, 904 East Caribbean Drive, Sunnyvale, CA 94089 USA; Present address: City of Hope, 1500 East Duarte Road, Rancho Cucamonga, CA 91010 USA

## Abstract

**Introduction:**

Accurate assessment of HER2 status is critical in determining appropriate therapy for breast cancer patients but the best HER2 testing methodology has yet to be defined. In this study, we compared quantitative HER2 expression by the HERmark™ Breast Cancer Assay (HERmark) with routine HER2 testing by immunohistochemistry (IHC) and fluorescence *in situ* hybridization (FISH), and correlated HER2 results with overall survival (OS) of breast cancer patients in a multicenter Collaborative Biomarker Study (CBS).

**Methods:**

Two hundred and thirty-two formalin-fixed, paraffin-embedded breast cancer tissues and local laboratory HER2 testing results were provided by 11 CBS sites. HERmark assay and central laboratory HER2 IHC retesting were retrospectively performed in a blinded fashion. HER2 results by all testing methods were obtained in 192 cases.

**Results:**

HERmark yielded a continuum of total HER2 expression (H2T) ranging from 0.3 to 403 RF/mm^2^ (approximately 3 logs). The distribution of H2T levels correlated significantly (*P* <0.0001) with all routine HER2 testing results. The concordance of positive and negative values (equivocal cases excluded) between HERmark and routine HER2 testing was 84% for local IHC, 96% for central IHC, 85% for local FISH, and 84% for local HER2 status. OS analysis revealed a significant correlation of shorter OS with HER2 positivity by local IHC (HR = 2.6, *P* = 0.016), central IHC (HR = 3.2, *P* = 0.015), and HERmark (HR = 5.1, *P* <0.0001) in this cohort of patients most of whom received no HER2-targeted therapy. The OS curve of discordant low (HER2 positive but H2T low, 10% of all cases) was aligned with concordant negative (HER2 negative and H2T low, HR = 1.9, *P* = 0.444), but showed a significantly longer OS than concordant positive (HER2 positive and H2T high, HR = 0.31, *P* = 0.024). Conversely, the OS curve of discordant high (HER2 negative but H2T high, 9% of all cases) was aligned with concordant positive (HR = 0.41, *P* = 0.105), but showed a significantly shorter OS than concordant negative (HR = 41, *P* <0.0001).

**Conclusions:**

Quantitative HER2 measurement by HERmark is highly sensitive, accurately quantifies HER2 protein expression and correlates well with routine HER2 testing. When HERmark and local HER2 results were discordant, HERmark more accurately predicted overall survival.

## Introduction

Overexpression of human epidermal growth factor receptor 2 (HER2) occurs in approximately 15 to 20% of primary breast carcinomas and is associated with poor prognosis [1,2]. HER2 tumor positivity is also a significant predictive factor for response to HER2-targeted therapies such as trastuzumab (Herceptin™), pertuzumab (Perjeta™), lapatinib (Tykerb™) or trastuzumab emtansine (T-DM1, Kadcyla™) [3-6]. Determination of the HER2 status for all invasive breast cancers at primary diagnosis is now the standard of care [7,8] and can be assessed by various HER2 testing methodologies. While slide-based HER2 assessments on formalin-fixed, paraffin-embedded (FFPE) breast cancer tissues are utilized, routine HER2 testing is subject to significant interlaboratory variation that may result in discrepant results in approximately 20% of routine HER2 testing in the community [7-10]. Reasons for discordance between laboratory HER2 results are complex and include differences in laboratory proficiencies and performances as well as interpretation of HER2 testing results. This has formed the basis of the expert American Society of Clinical Oncology (ASCO) and the College of American Pathologists (CAP) panel in 2007 to develop guidelines to improve the accuracy of HER2 testing in breast cancer [7]. Recently published reports continue to show lack of concordance for HER2 results between laboratories despite significant emphasis and progress made to standardize routine HER2 testing post publication of the ASCO/CAP guidelines for HER2 testing in 2007 [11-13]. Since then, clarifications and updates to the ASCO/CAP HER2 testing guidelines have been issued, and ASCO and CAP convened to conduct a formal and comprehensive review and revised the guidelines in 2013 [8]. The main objective of HER2 testing remains to accurately determine which patients may benefit from HER2-based targeted therapies.

The HERmark™ Breast Cancer Assay (HERmark, Monogram Biosciences, South San Francisco, CA, USA) is a validated novel HER2 testing method that provides accurate quantification of HER2 protein in FFPE tissue samples [14,15]. The HERmark assay uses a dual-antibody, proximity-based immunoassay approach, the VeraTag™ technology (Monogram Biosciences), to make precise and quantitative measurement of total HER2 protein expression (H2T) with greater sensitivity and specificity than immunohistochemistry (IHC), and provides a continuum of H2T values over approximately a 1,000-fold dynamic range in human breast cancers and cell lines [15]. The utilization of capillary electrophoresis for HER2 signal quantification in the HERmark assay yields results that are independent of an observer’s subjective interpretation of HER2 signal intensity. A clinical study showed that when breast cancer HER2 expression was measured using the HERmark assay, breast cancer patients with advanced disease whose tumors were HER2 positive by fluorescence *in situ* hybridization (FISH) but H2T low by HERmark had similar outcomes (as measured by time to progression, following treatment with trastuzumab) as those patients whose cancers were FISH negative and H2T low, thus identifying HER2 FISH-positive tumors with a poorer response to trastuzumab, relative to those tumors which were both HER2 positive by FISH and H2T high by HERmark [16]. These data suggest that HERmark may serve as a novel alternative for quantitative HER2 assessment and provide added value to routine HER2 testing. The aims of the current study were to evaluate the concordance of HER2 results between HERmark and routine HER2 testing methods, and to correlate the results obtained by various HER2 methods with overall survival (OS) of breast cancer patients in a multicenter Collaborative Biomarker Study (CBS) involving 11 study sites.

## Methods

### Patient population

This retrospective, multicenter Collaborative Biomarker Study included women who had tumor tissues from routine surgical excision of invasive breast cancer between January 2000 and May 2005 from the following study sites: Sarah Cannon Research Institute (Nashville, TN, USA)/Tennessee Oncology, PLLC, (Nashville, TN, USA), Dartmouth Hitchcock Medical Center (Lebanon, NH, USA), Texas Oncology Bedford (Bedford, TX, USA), Texas Oncology and Medical City (Dallas, TX, USA), St. Jude Heritage Medical Group (Fullerton, CA, USA), the Center for Cancer and Hematologic Disease (Cherry Hill, NJ, USA), Monroe Medical Associates (Harvey, IL, USA), Swedish American Regional Medical Center (Rockford, IL, USA), the West Clinic (Memphis, TN, USA), Arlington Cancer Center (Arlington, TX, USA), and Wilshire Oncology Medical Group (Rancho Cucamonga, CA, USA). Institutional review board (IRB) approval for the study was conducted by the Western Institutional Review Board (WIRB, Olympia, WA, USA) for our sponsor approval and site IRB approvals. The study was granted a waiver for informed consents and a waiver for authorization under HIPAA, which we specifically requested due to the nature of the study and likelihood that many of the patients whose tumors we studied were now deceased or lost to follow-up. The WIRB determined that this study qualifies for a waiver of consent under 45 CFR 46.116(d). The Board also approved our request for a waiver of authorization for use and disclosure of protected health information (PHI) for the study. Tissue samples and clinical data were anonymized. A total of 232 FFPE breast cancer blocks were collected by the 11 CBS study sites with a median of 22 (range 5 to 27) cases per site. The study was designed to enroll approximately 50% cases with positive HER2 status and 50% with negative HER2 status as determined by study sites based on local HER2 testing. Freshly cut 5-micron unstained tissue sections were prepared on positively charged glass slides by study sites and sent to Monogram Biosciences for HERmark assay and central HER2 IHC retest in a blinded fashion. Patient-deidentified pathology reports were reviewed and clinicopathological data, including demographics, tumor characteristics, hormone receptor status, treatment (including HER2-targeted therapy received), prior HER2 testing results, and OS data, were collected by study sites, and sent to Monogram Biosciences following the site’s receipt of each patient’s HERmark result.

### The HERmark™ Breast Cancer Assay

Total HER2 protein expression (H2T) was quantified using the HERmark assay as previously described [14,15,17]. Briefly, H2T was detected through the proximity-based release of a fluorescent tag conjugated via a cleavable thioether tether, to a monoclonal antibody directed against the cytoplasmic domain of HER2 (Ab8, LabVision, part of Thermo Fisher Scientific, Waltham, MA, USA). The antibody was paired with a biotinylated second antibody directed against the C-terminus of HER2 (Ab15, LabVision, part of Thermo Fisher Scientific). Upon illumination with red light, the photosensitizer molecule linked to the second biotinylated Ab15 liberates singlet oxygen that cleaves any neighboring thioether bonds, thus releasing VeraTag™ reporters in close proximity. Reporter signal quantified by capillary electrophoresis was normalized to invasive tumor area on the FFPE tissue section. The final value for H2T expression (relative fluorescence (RF)/mm^2^ tumor) was calculated as (RF concentration) × (illumination buffer volume)/(tumor area). A total of 194 (84%, 194/232) collected cases were determined to have adequate invasive breast cancer tissues and yielded valid H2T results by HERmark assay. The HERmark assay was performed and H2T results were determined, blinded to local HER2 results and case report form data including clinical outcomes and treatment information. The continuous H2T results were categorized as HERmark negative, HERmark equivocal, and HERmark positive with predefined H2T analytical cutoff values (<10.5, 10.5 to 17.8, and >17.8 RF/mm^2^, respectively [17]). A pre-defined, published HERmark clinical cutoff (13.8 RF/mm^2^ [16]) was also used to define H2T levels as H2T low and H2T high in OS analysis.

### Central HER2 immunohistochemistry (IHC) retesting

Central laboratory HER2 IHC retest was performed at the Center for Molecular Biology and Pathology (CMBP, Laboratory Corporation of America, Inc., Research Triangle Park, NC, USA), using the HercepTest™ (DAKO, Glostrup, Denmark). A total of 192 (99%) of the 194 cases that had H2T results yielded valid HER2 IHC results. Central HER2 IHC retest was performed and analyzed blinded to HERmark and local HER2 results. The IHC staining intensity was scored as 0, 1+, 2+, or 3+ and categorized as HER2 IHC negative, equivocal, or positive according to the ASCO/CAP guideline recommendations for HER2 testing [7].

### Local HER2 testing results

Each study site collected and reported HER2 IHC (defined as local IHC in this study) and/or HER2 FISH (defined as local FISH) results for each case. In addition, each study site reported a final clinical HER2 status (negative, equivocal, or positive, and defined as local HER2 status or investigator-determined HER2 status) based on the combination of all available local HER2 results for each case. Local IHC was reported as 0, 1+, 2+, or 3+ staining as well as IHC negative, equivocal, or positive as determined by study sites. Local FISH was reported as HER2 amplified or HER2 non-amplified only, and information on HER2/CEP17 ratio was not available for this study. No FISH equivocal case was submitted. Local FISH results were available in 67 (35%) of the 194 cases that had H2T results.

### Statistical analysis

The agreement between HERmark and HER2 IHC, HER2 FISH- or investigator-determined HER2 status was analyzed according to the number of tumors defined for each category divided by the total number of study patients. Differences in continuous H2T values among various categorical HER2 subgroups defined by routine HER2 testing methods were analyzed using the Mann-Whitney test or the Jonckheere-Terpstra test. Overall concordance that included all HER2 categories as well as positive/negative concordance that excluded equivocal HER2 cases was analyzed in accordance with the ASCO/CAP HER2 testing guidelines [7]. Kappa statistics were used to evaluate concordance between HERmark and routine HER2 testing methods. OS was defined as the time from initial diagnosis of invasive breast cancer to death or censor. Estimates of survival were based on the Kaplan-Meier (KM) method and statistical significance of differences was evaluated by the log-rank test.

## Results

### Patient and tumor characteristics

Patient clinicopathological characteristics are summarized in Table 1. A total of 194 samples that had quantitative HER2 measurements by HERmark were included for analysis. Median age was 51 years (range 27 to 84 years). The majority of tumor samples were of primary breast cancers (96%), invasive ductal carcinoma histology (89%), moderately to poorly differentiated (73%), stage I or II at initial diagnosis (71%), and hormone receptor status (estrogen receptor (ER)/progesterone receptor (PR)) positive (73%). Local HER2 status was reported by the study sites as 57% HER2 negative, 43% HER2 positive and 1% HER2 equivocal. Only 10% of patients were reported to have received targeted HER2 therapy (trastuzumab and/or lapatinib, Table 1). This retrospective study included women who had tumor tissues from routine surgical excision of invasive breast cancer between January 2000 and May 2005. HER2-targeted therapy was not FDA approved outside of clinical trials for neoadjuvant or early-stage breast cancer therapy during the study period. The median follow-up time was 67.1 months (range 14.8 to 302.8 months).

**Table 1:** **Clinicopathological characteristics of the study population**

**Parameter**	**No.**	**% (range)**
**Sample size**	194	
**Median length of follow-up (months)**	193	67.1 (14.8 - 302.8)
**Median age (years)**		51 (27 - 84)
<40	21	11%
40-49	66	34%
50-59	50	26%
≥60	57	29%
**Menopausal status**		
Premenopausal	75	39%
Perimenopausal	8	4%
Postmenopausal	96	49%
Not reported	15	8%
**Tissue source**		
Primary breast	187	96%
Other^*^	7	4%
**Tumor histology**		
Invasive ductal carcinoma	172	89%
Invasive lobular carcinoma	14	7%
Other histologic type^**^	8	4%
**Median tumor size (cm)**	185	2.1 (0.4 - 14)
Not reported	9	
**Tumor grade**		
Grade 1 (well)	17	9%
Grade 2 (moderate)	49	25%
Grade 3 (poor)	93	48%
Not reported	35	18%
**Stage at initial diagnosis**		
I	46	24%
II	91	47%
III	40	21%
IV	13	7%
Not reported	4	2%
**Nodal status at initial diagnosis**		
Node positive	89	46%
Node negative	66	34%
Not reported	39	20%
**HER2 status (reported)**		
Positive	83	43%
Negative	110	57%
Equivocal	1	1%
**HER2 IHC (reported)**		
3+	73	38%
2+	30	16%
*IHC 2+/FISH positive*	*2*	*7%*
*IHC 2+/FISH negative*	*18*	*60%*
*IHC 2+/FISH not reported*	*10*	*33%*
1+	32	17%
0	56	29%
**HER2 FISH (reported)**		
Positive	23	36%
Negative	41	64%
**Hormone receptor (ER/PR) status**		
Positive	141	73%
Negative	53	27%
**ER and PR status**		
ER (+), PR (+)	110	57%
ER (+), PR (−)	30	15%
ER (−), PR (+)	1	1%
ER (−), PR (−)	53	27%
**HER2 targeted therapy** ^**#**^		
No	174	90%
Yes	20	10%

^*^Other tissue sources: skin, supraclavicular, sentinental lymph node, axillary lymph node, ovary, lung, chest wall; ^**^other histologic type: ‘invasive mammary’ (5), breast adenocarcinoma (1), infiltrating mucinous, adenocarcinoma (1), and metastatic carcinoma (1); ^#^trastuzumab- and/or lapatinib-containing therapy. ER, estrogen receptor; FISH, fluorescence *in situ* hybridization; HER2, human epidermal growth factor receptor 2; IHC, immunohistochemistry; PR, progesterone receptor.

### Comparison of HER2 results and various testing methods

**Figure 1:**
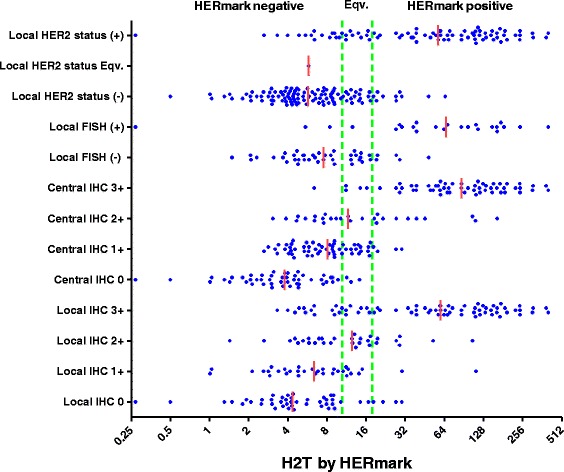
**Distribution of quantitative total HER2 expression (H2T) in routine HER2 tests.** HERmark equivocal (Eqv.) zone is defined within the two green vertical lines. Short vertical red line indicates the median of a H2T distribution. FISH, fluorescence *in situ* hybridization; HER2, human epidermal growth factor receptor 2; H2T, quantitative total HER2 expression by HERmark; IHC, immunohistochemistry.

The distribution of quantitative total HER2 expression (H2T) was compared with categorical results of routine HER2 tests (Figure 1). Quantitative H2T values ranged from 0.3 to 403 RF/mm^2^ on a continuum for the entire cohort. In comparison with local IHC, the H2T levels ranged from 0.5 to 30.1 (median 4.4) for IHC 0, from 1.0 to 112 (median 6.4) for IHC 1+, from 1.4 to 105 (median 12.5) for IHC 2+, and from 3.3 to 403 (median 59.6) for IHC 3+. Higher H2T levels were significantly associated with stronger IHC staining intensity in the four subgroups by local IHC testing (*P* <0.0001, Jonckheere-Terpstra test). For central IHC retest cases, the H2T levels ranged from 0.3 to 14.3 (median 3.8) for IHC 0, from 2.6 to 30.1 (median 8.1) for IHC 1+, from 3.1 to 163 (median 11.7) for IHC 2+, and from 6.4 to 403 (median 86.5) for IHC 3+. Higher H2T levels were significantly associated with stronger IHC staining intensity in the four central IHC subgroups (*P* <0.0001, Jonckheere-Terpstra test). In comparison with local FISH, the H2T levels ranged from 1.5 to 48.4 (median 7.6) for FISH negative and from 0.3 to 403 (median 65.7) for FISH positive. In comparing H2T levels in FISH-negative versus FISH-positive groups, we observed significantly higher H2T in the FISH-positive group (*P* <0.0001, Mann-Whitney test). For local HER2 status, the H2T levels ranged from 0.5 to 65.0 (median 5.7) for HER2 negative and from 0.3 to 403 (median 57.4) for HER2 positive. Higher H2T levels were significantly associated with local HER2 status positive (*P* <0.0001, Mann-Whitney test). The sole case of local HER2 status equivocal had an H2T value of 5.8 and therefore was categorized as HERmark negative.

The comparison between HERmark and routine HER2 tests is presented in Table 2. The overall concordance of all three categories (negative, equivocal, and positive) between HERmark and routine HER2 testing was 68% (kappa 0.475) for local IHC, 69% (kappa 0.481) for central IHC, and 73% (kappa 0.510) for local HER2 status. Analysis of overall concordance between HERmark and local FISH was not performed because there was no FISH equivocal case reported by study sites. When equivocal cases were excluded from all HER2 results, the concordance of positive and negative values between HERmark and routine HER2 testing was 84% (kappa 0.676) for local IHC, 96% (kappa 0.914) for central IHC, 85% (kappa 0.705) for local FISH, and 84% (kappa 0.682) for local HER2 status.

**Table 2:** **Concordance of HERmark with local HER2 IHC, central HER2 IHC, local HER2 FISH, and local clinical HER2 status**

			**Local HER2 IHC**								**Central HER2 IHC**								**Local HER2 FISH**								**Local clinical HER2 status**					
			**Negative**		**Equivocal**		**Positive**		**Total IHC**		**Negative**		**Equivocal**		**Positive**		**Total IHC**		**Non-amplified**		**Amplified**		**Total FISH**		**Negative**		**Equivocal**		**Positive**		**Total HER2**	
			**N**	**%**	**N**	**%**	**N**	**%**	**N**	**%**	**N**	**%**	**N**	**%**	**N**	**%**	**N**	**%**	**N**	**%**	**N**	**%**	**N**	**%**	**N**	**%**	**N**	**%**	**N**	**%**	**N**	**%**
HERmark	Negative		69	71%	14	14%	14	14%	97	51%	86	86%	13	13%	1	1%	100	52%	27	90%	3	10%	30	45%	83	83%	1	1%	16	16%	100	52%
HERmark	Equivocal		10	38%	9	35%	7	27%	26	14%	17	71%	5	21%	2	8%	24	13%	11	92%	1	8%	12	18%	17	65%	0	0%	9	35%	26	13%
HERmark	Positive		9	13%	7	10%	52	76%	68	36%	4	6%	23	34%	41	60%	68	35%	5	20%	20	80%	25	37%	10	15%	0	0%	58	85%	68	35%
		Total	88	46%	30	16%	73	38%	191	100%	107	56%	41	21%	44	23%	192	100%	43	64%	24	36%	67	100%	110	57%	1	1%	83	43%	194	100%
Overall concordance			68%, (69 + 9 + 52)/191								69%, (86 + 5 + 41)/192								NA^#^						73%, (83 + 0 + 58)/194							
Kappa (CI 95%), overall			0.475 (0.373 to 0.578); weighted Kappa = 0.545								0.481 (0.386 to 0.576); weighted Kappa = 0.631														0.510 (0.409 to 0.610); weighted Kappa = 0.583							
Concordance, excluding Eqv.^*^			84%, (69 + 52)/(69 + 9 + 14 + 52)								96%, (86 + 41)/(86 + 4 + 1 + 41)								85%, (27 + 20)/(27 + 5 + 3 + 20)						84%, (83 + 58)/(83 + 10 + 16 + 58)							
Kappa (CI 95%), excluding Eqv.			0.676 (0.550 to 0.797)								0.914 (0.841 to 0.988)								0.705 (0.516 to 0.893)						0.682 (0.570 to 0.794)							

^*^Equivocal (Eqv.) cases from both tests were excluded; ^#^NA, overall concordance was not calculated for 2 x 3 table. Central HER2 IHC retesting was performed retrospectively and central HER2 status was defined per ASCO/CAP guidelines for HER2 testing [7]. The results of local HER2 testing were reported by participating study sites based on local IHC and/or local FISH. Percentages may not add up to 100% due to rounding. CI, confidence interval; FISH, fluorescence *in situ* hybridization; HER2, human epidermal growth factor receptor 2; IHC, immunohistochemistry.

### Kaplan-Meier overall survival analyses by HER2 status as stratified by different HER2 testing methods

**Figure 2:**
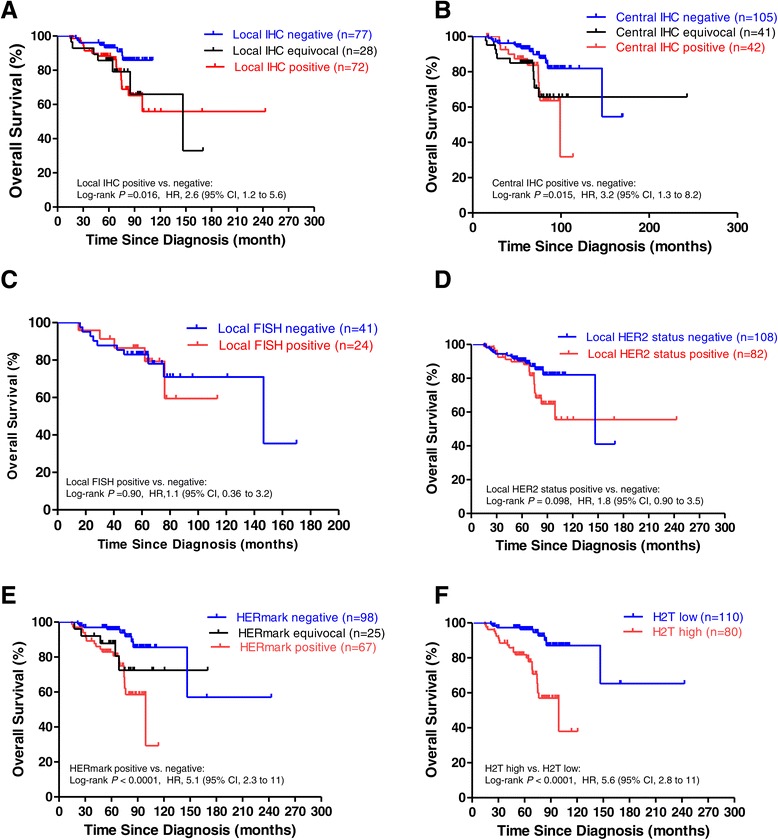
**Kaplan-Meier overall survival analyses by HER2 status. (A)** Local HER2 IHC, **(B)** central HER2 IHC, **(C)** local HER2 FISH, **(D)** local HER2 status, **(E)** HERmark HER2 status, and **(F)** quantitative total HER2 (H2T) levels. The results of local HER2 tests were reported by participating study sites. Central HER2 IHC retest was performed retrospectively and categorized per ASCO/CAP guidelines for HER2 testing [7]. HERmark HER2 status was categorized as HERmark negative, HERmark equivocal, or HERmark positive by predefined H2T analytical cutoff values [17]. H2T low and H2T high were defined by a predetermined H2T clinical cutoff [16]. CI, confidence interval; FISH, fluorescence *in situ* hybridization; HER2, human epidermal growth factor receptor 2; H2T, quantitative total HER2 expression by HERmark; HR, hazard ratio; IHC, immunohistochemistry.

The Kaplan-Meier overall survival (OS) analysis was performed on cases that had HER2 testing results and available survival data in 177 cases with local IHC, 188 cases with central IHC, 65 cases with local FISH, 190 cases with local HER2 status and 190 cases with HERmark (Figure 2). Local IHC positive (IHC 3+) was associated with a significantly shorter OS compared with local IHC negative (IHC 0 to 1+) (Figure 2A: hazard ratio (HR) = 2.6; 95% confidence interval (CI), 1.2 to 5.6; *P* = 0.016). OS for local IHC equivocal (IHC 2+) was similar to that of local IHC positive (Figure 2A: HR = 1.0; 95% CI, 0.43 to 2.6; *P* = 0.92) and showed a trend of shorter OS compared with local IHC negative (HR = 2.7; 95% CI, 0.82 to 9.2; *P* = 0.10). Similarly, central IHC positive was associated with a significantly shorter OS compared with central IHC negative (Figure 2B: HR = 3.2; 95% CI, 1.3 to 8.2; *P* = 0.015). OS in central IHC equivocal was similar to that of central IHC positive (Figure 2B: HR = 0.96; 95% CI, 0.41 to 2.3; *P* = 0.93) and showed a trend (near statistically significant) of shorter OS compared with the central IHC negative (HR = 2.5; 95% CI, 1.0 to 6.5; *P* = 0.051). Local FISH positive did not show significant differences in OS compared with local FISH negative (Figure 2C: HR = 1.1; 95% CI, 0.36 to 3.2; *P* = 0.90). Investigator-determined HER2-positive status correlated with a trend of shorter OS compared with HER2-negative status (Figure 2D: HR = 1.8; 95% CI, 0.90 to 3.5 *P* = 0.098). HERmark positive was associated with a significantly shorter OS compared with HERmark negative (Figure 2E: HR = 5.1; 95% CI, 2.3 to 11; *P* <0.0001). OS of HERmark equivocal was similar to that of HERmark positive (Figure 2E: HR = 0.62; 95% CI, 0.26 to 1.5; *P* = 0.29), and showed a trend of shorter OS compared with HERmark-negative cases (HR = 3.2, 95% CI, 0.82 to 12; *P* = 0.093). H2T high (H2T >13.8) was associated with a significantly shorter OS compared with H2T low (Figure 2F: HR = 5.6, 95% CI, 2.8 to 11; *P* <0.0001). When OS analysis for H2T levels was restricted to patients who had not received any HER2-targeted therapy, similar results were observed, showing that H2T high status was associated with a significantly shorter OS compared with H2T low status (HR = 4.7, 95% CI, 1.7 to 13; *P* = 0.003).

**Figure 3:**
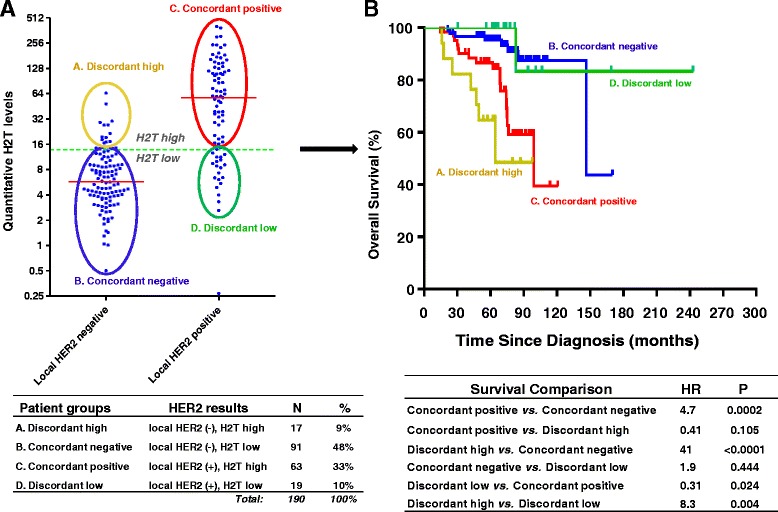
**Overall survival and HER2 status as stratified by local HER2 status and H2T levels. (A)** H2T distribution by local HER2 status. **(B)** Kaplan-Meier overall survival analyses for corresponding HER2-H2T concordant and discordant groups. Short horizontal red line in **(A)** indicates the median of a H2T distribution. HER2, human epidermal growth factor receptor 2; H2T, quantitative total HER2 expression by HERmark; HR, hazard ratio; *P*, the *P* value of log-rank test.

The OS correlation between local HER2 status and H2T levels was further explored by plotting the distribution of quantitative H2T levels versus the corresponding local HER2 status and by comparing outcomes in four groups defined by concordant or discordant results using the HERmark H2T clinical cutoff (H2T = 13.8, Figure 3). The distribution of high or low H2T values in local HER2-negative and HER2-positive groups overlapped and resulted in four subgroups of H2T distribution (Figure 3A): (1) patients who were HER2 status negative and H2T low (concordant negative, N = 91, 48%), (2) patients who were HER2 status positive and H2T high (concordant positive, N = 63, 33%), (3) patients who were HER2 status positive but H2T low (discordant low, N = 19, 10%) and (4) patients who were HER2 status negative but H2T high (discordant high, N = 17, 9%).

Differences in patient clinicopathological characteristics of the concordant and discordant groups are summarized in Table 3. Compared to the concordant negative, cases of the concordant positive were significantly associated with higher tumor grade, higher stage at initial diagnosis, positive nodal status, and negative hormone receptor status. However, no statistically significant differences in the clinicopathological characteristics were found between the two groups that were discordant between local HER2 status and H2T high/low.

**Table 3:** **Clinicopathologic characteristics of HER2 status-HERmark concordant and discordant groups**

	**Concordant negative**		**Concordant positive**		**Discordant low**		**Discordant high**	
	**HER2 -, H2T low**		**HER2 +, H2T high**		**HER2 +, H2T low**		**HER2 -, H2T high**	
**Parameter**	**No.**	**%**	**No.**	**%**	**No.**	**%**	**No.**	**%**
**Tumor size (cm)**								
<2	45	51%	30	49%	9	47%	6	43%
>2	43	49%	31	51%	10	53%	8	57%
**Tumor grade**								
Grade 1 and Grade 2	41	54%	10	19%^*^	6	40%	8	62%
Grade 3	35	46%	43	81%	9	60%	5	38%
**Stage at initial diagnosis**								
I and II	73	83%	40	65%	14	74%	8	47%
III and IV	15	17%	22	35%^*^	5	26%	9	53%
**Nodal status at diagnosis**								
Node negative	40	55%	15	28%	7	47%	3	25%
Node positive	33	45%	38	72%	8	53%	9	75%
**Hormone receptor (ER/PR) status**								
Negative	19	21%	23	37%^*^	6	32%	4	24%
Positive	72	79%	40	63%^*^	13	68%	13	76%

^*^ *P* <0.05. Fisher exact test was performed for each parameter between two concordant groups, or, between two discordant groups. ER, estrogen receptor; HER2, human epidermal growth factor receptor 2; H2T, quantitative total HER2 expression by HERmark; PR, progesterone receptor.

Cox proportional hazards multivariate analyses were then conducted to evaluate correlation of HERmark subgroups with OS in the context of clinicopathological variables that showed significant differences between concordant negative and concordant positive (Table 4). Two different sets of cut points were used to define HERmark subgroups for the analyses. In Model A, the ‘analytical cut points’, that is <10.5, 10.5 to 17.8 and >17.8 RF/mm^2^, were established from HERmark validation study in which H2T values were compared with central HER2 IHC/*in situ* hybridization (ISH) results [17]. The analytical cut points, which define HERmark negative, HERmark equivocal, and HERmark positive, are often used to determine HERmark HER2 status and to compare routine HER2 status by IHC/ISH with HERmark. In Model B, the ‘clinical cutoff’, that is 13.8 RF/mm^2^, was established from a HERmark clinical study in which an optimal H2T cut point was established for the purpose for identifying better responders in a clinical cohort of breast cancer patients treated with anti-HER2 therapy [16]. The clinical cut point, which defines H2T high and H2T low, is often used to correlate HERmark results with patient outcome in clinical studies. In both Model A and Model B, HER2 subgroups by HERmark were identified as independent variables that correlate with OS. Patients in HERmark-positive or H2T-high group exhibited significant shorter overall survival compared with patients in HERmark-negative or H2T-low group, respectively. None of the other clinicopathological variables (grade, stage, nodal status, and hormonal status) was identified as an independent variable that significantly correlated with OS.

**Table 4:** **Multivariate Cox proportional hazards ratios for association between HER2 measurements by HERmark, selected clinicopathological variables and overall survival**

	**Overall survival**	
	**HR**	***P*** **value**
**Model A**		
HERmark positive vs. negative^*^	3.1	0.034
Grade 3 vs. 1 or 2	2.0	0.26
Stage III or IV vs. I or II	0.93	0.89
Node positive vs. negative	1.7	0.38
Hormone receptor positive vs. negative	0.43	0.071
**Model B**		
H2T high vs. low^#^	3.9	0.0061
Grade 3 vs. 1 or 2	1.7	0.30
Stage III or IV vs. I or II	0.91	0.84
Node positive vs. negative	1.9	0.26
Hormone receptor positive vs. negative	0.54	0.15

^*^HERmark status based on HERmark analytical cutoffs in Model A: HERmark positive - H2T >17.8 relative fluorescence (RF)/mm^2^; HERmark negative - H2T <10.5 RF/mm^2^. ^#^HER2 levels based on HERmark clinical cutoff in Model B: H2T high - H2T >13.8 RF/mm^2^; H2T low- H2T ≤13.8 RF/mm^2^. HER2, human epidermal growth factor receptor 2; H2T, quantitative total HER2 expression by HERmark; HR, hazard ratio.

Next, we performed Kaplan-Meier analysis to evaluate OS for the patients in discordant high group or the discordant low group in comparison with patients in either the concordant-positive group or the concordant-negative group (Figure 3B). The concordant-positive group was associated with a significantly shorter OS compared with the concordant-negative group (HR = 4.7, 95% CI, 2.1 to 11; *P* = 0.0002). The discordant-high group generated an OS curve that tracked with the concordant-positive group (HR = 0.41, 95% CI, 0.14 to 1.2; *P* = 0.105), but showed a significantly shorter OS compared with the concordant-negative group (HR = 41, 95% CI, 8.1 to 212; *P* <0.0001). The discordant-low group yielded an OS curve that tracked with the concordant-negative group (HR = 1.9, 95% CI, 0.37 to 9.9; *P* = 0.444), but showed a significantly longer OS compared with the concordant-positive group (HR = 0.31, 95% CI, 0.11 to 0.86; log-rank *P* = 0.024). In comparing the two discordant groups, the discordant-high group was associated with a significantly shorter OS compared with the discordant-low group (HR = 8.3, 95% CI, 1.9 to 35; *P* = 0.0042). These results indicate that in the cases with discordance in local HER2 status versus H2T results, the expected overall survival correlates with HERmark results rather than with local HER2 status.

## Discussion

Despite efforts made in the past decade to improve the accuracy and standardization of HER2 testing, discordance in HER2 results between laboratories and testing methodologies persists. The neoadjuvant GeparQuattro trial reported a rather high rate of discordance of 27% between central and local evaluation of HER2 status [12], which is similar to previous reports of inaccurate local HER2 results in the NSABP B-31 [18] and the NCCTG N9831 studies [10,19]. A round-robin study reported a pre-adjudication discordance rate of 8% for HER2 status (both IHC and FISH) among three expert pathologists from three central laboratories [13], similar to the results from an international HER2 proficiency group study performed among five central laboratories [20]. The main objectives of the current study were to compare HER2 results between a novel HER2 test (HERmark) and routine HER2 tests (IHC and FISH), and to correlate HER2 results by various HER2 testing methods with overall survival of breast cancer patients from a multicenter Collaborative Biomarker Study.

The quantitative HERmark assay demonstrated a broad continuum (approximately 3 logs) of HER2 expression levels in breast cancer samples in this study. While distribution of H2T values correlated significantly with all routine HER2 testing methods, higher concordance was observed between HERmark and central IHC retest as compared to local HER2 tests. This is consistent with previous reports noting higher discordance between local HER2 testing and central HER2 testing relative to discordance among central laboratories [12,18,19]. Thus, the HERmark assay may supplement existing routine HER2 testing in the ‘real-world’ setting as an accurate central HER2 test alternative, particularly for those cases with uncertainty in routine HER2 results, for example, HER2 equivocal cases and HER2 results that show inconsistencies with other clinical and pathological parameters. In addition, the highly sensitive HERmark assay (approximately seven to ten times more sensitive than HER2 IHC [14,15]) may serve as a reliable tool to quantify HER2 expression levels in HER2 non-overexpressing breast cancers where routine HER2 testing may not be optimized to measure lower levels of HER2 protein. Interesting data from the NSABP B31 study suggest that HER2 non-overexpressing breast cancer may benefit from targeted HER2 therapy [21]. For HER2 expression that does not meet the threshold for HER2-positive disease, enrollment into prospective clinical trials is encouraged, such as NCY01275677 (NSABP B47) that aims to address the benefit of adjuvant HER2-targeted therapies in tumors with a lower level of HER2 expression. More accurate and quantitative measurement of lower HER2 expression levels may also be helpful for ongoing and planned trials of new anti-HER2 therapies, such as investigational anti-HER2 monoclonal antibodies such as margetuximab and anti-HER2 vaccines targeting HER2 non-overexpressing breast cancer patients [22-24].

We evaluated HER2 status, stratified by routine HER2 testing and HERmark, as a prognostic factor for OS. Most patients in this study did not receive targeted HER2 therapy, and thus, positive HER2 status was expected to correlate with shorter OS. Accordingly, the results showed that HER2 positivity by local IHC, central IHC, or HERmark was significantly associated with a shorter OS compared with HER2 negativity. However, local FISH did not show significant difference in OS between FISH-positive and FISH-negative groups. Possible explanations may include small sample size and selection bias because only a portion of the cases had undergone HER2 FISH testing. Local HER2-positive status, which is used routinely in clinical practice to identify patients with HER2-positive tumors for targeted HER2 therapies, showed a trend, but not statistically significant, of shorter OS (HR = 1.8, *P* = 0.098). In contrast, H2T levels, as predefined by a published HERmark clinical cutoff [16], demonstrated a significantly shorter OS in H2T high compared to H2T low (HR = 5.6, *P* <0.0001).

To further explore the difference in OS between local HER2 status and HERmark H2T results, we performed Kaplan-Meier analysis on four HER2-HERmark subgroups: concordant negative (HER2 negative and H2T low), concordant positive (HER2 positive and H2T high), discordant low (HER2 positive and H2T low), and discordant high (HER2 negative and H2T high), (Figure 3). Each discordant group comprises a significant portion (9 to 10%) of the entire cohort. As expected, the concordant-positive group was associated with a significantly shorter OS when compared with the concordant-negative group. Where the results of local HER2 status were discordant with those of HERmark, as in the two discordant groups, the OS curves were significantly aligned with those of HERmark results, but not with the local HER2 status. The results suggest that within the discordant groups, the HERmark results appear to be a better predictor of OS in this clinical cohort. We attempted to explore potential underlying reasons for these differences and compared clinicopathological characteristics of the two discordant groups (Table 3). While concordant positives were significantly associated with higher tumor grade, more advanced stage at initial diagnosis, more positive nodal status, and higher rate of hormone receptor negativity, no significant differences were found in these clinicopathological characteristics between the two discordant groups. In Cox proportional hazards multivariate analyses (Table 4), HER2 expression was identified as the only independent variable that significantly correlates with OS. Although the sample sizes of the discordant groups were relatively small, the difference in OS does not appear to be due to differences in the clinicopathological factors, but likely due to the ‘miscall’ made by local HER2 status. If these findings are confirmed with further studies, the clinical implications can be significant. Importantly, the results suggest that 10% of breast cancer patients may be misclassified in routine HER2 testing as false negative, leading to denial of HER2-targeted therapies for patients that could benefit from it. On the other hand, patients misclassified in routine HER2 testing as false positive (approximately 10%) could lead to the prescribing and administration of costly and potentially toxic ineffective treatment with targeted HER2 therapies. Although the total number of patients selected for anti-HER2 treatment would not change with this scenario, HERmark may offer more accurate selection of breast cancer patients for targeted HER2 therapy in a significant proportion (approximately 20%) of patients. Obviously, cost and turnaround time implications of additional HER2 tests using a method like HERmark should be further evaluated. Recently, a study of cost-effectiveness for expanded HER2 reflex testing was reported [25]. The analysis showed that retesting patients who are IHC 0, IHC1+, or FISH-negative is projected to be a cost-effective clinical strategy. The expanded reflex testing allows for a second opportunity to measure HER2 status accurately, correcting both handling errors and testing inconsistency. Similarly, we believe that selective HER2 retest by HERmark for patients who had discordant or inconclusive HER2 status by IHC/ISH would also be a cost-effective clinical strategy, which warrants further investigation.

The strengths of this study include the multicenter setting which represents ‘real-world’ routine HER2 testing in the field and thus, is relevant to clinical practice. Evaluation of various HER2 results, including those of routine local and central HER2 tests coupled with the novel HERmark HER2 quantification, offers a comprehensive comparison among various HER2 testing methods. Despite the heterogeneous therapies in the clinical patient cohort from various study sites, the majority of patients did not receive HER2-targeted therapy and thus OS is a relatively reliable clinical outcome in such a setting. The study has several limitations. The retrospective nature of this biomarker study carries inherent heterogeneities that exist not only in the tumor specimens evaluated but also in patient selection, HER2 testing methods, and treatments. The study was designed to include approximately 50% HER2-positive and 50% HER2-negative cases for effective comparison of HER2 results by various testing methods, and thus does not represent HER2 status in a general population of breast cancer patients in which the expected rate of HER2 positivity is approximately 15%. The number of cases in each of the two discordant groups is relatively small. Thus, the issues with relatively small groups in a relatively skewed patient population should be addressed in future studies. Since most patients in this study did not receive targeted HER2 therapy, further studies are also needed to investigate anti-HER2 response in patients of the discordant groups.

## Conclusions

The novel HERmark assay offers highly sensitive and accurate quantification of HER2 protein expression that has demonstrated excellent concordance with central HER2 testing and better correlation as a prognostic factor in OS as compared with ‘real-world’ local HER2 status in a multicenter clinical cohort of breast cancer patients. The HERmark assay may reclassify 10% of false-negative patients by conventional tests as truly positive and 10% who are currently testing false positive by conventional tests as negative. Thus, a resultant change in therapy for 20% of patients may improve the outcomes for both HER2-negative and -positive cohorts. Further clinical studies are warranted to confirm these findings within well-controlled clinical cohorts and clinical trials.

## Abbreviations

CI: confidence interval
ER: estrogen receptor
FFPE: formalin-fixed, paraffin-embedded
FISH: fluorescence *in situ* hybridization
HER2: human epidermal growth factor receptor 2
H2T: quantitative total HER2 expression by HERmark
HR: hazard ratio
IHC: immunohistochemistry
ISH: *in situ* hybridization
OS: overall survival
PR: progesterone receptor
RF: relative fluorescence
